# BAY 87–2243, a novel inhibitor of hypoxia-induced gene activation, improves local tumor control after fractionated irradiation in a schedule-dependent manner in head and neck human xenografts

**DOI:** 10.1186/1748-717X-9-207

**Published:** 2014-09-19

**Authors:** Linda Helbig, Lydia Koi, Kerstin Brüchner, Kristin Gurtner, Holger Hess-Stumpp, Kerstin Unterschemmann, Michael Baumann, Daniel Zips, Ala Yaromina

**Affiliations:** OncoRay – National Center for Radiation Research in Oncology, Medical Faculty Carl Gustav Carus, TU Dresden, Dresden, Germany; Department of Radiation Oncology, University Hospital Carl Gustav Carus, TU Dresden, Dresden, Germany; German Cancer Consortium (DKTK), Dresden and German Cancer Research center (DKFZ), Heidelberg, Germany; Institute of Radiooncology Helmholtz-Zentrum Dresden-Rossendorf, Dresden, Germany; Global Drug Discovery, Bayer Pharma AG, Berlin and Wuppertal, Germany; Radiation Oncology, Eberhard Karls University Tübingen, Tübingen, Germany; German Cancer Consortium (DKTK), Tübingen, Germany; Department of Radiation Oncology (MAASTRO Lab), GROW – School for Oncology and Developmental Biology, Maastricht University Medical Centre, Maastricht, The Netherlands

**Keywords:** HIF pathway inhibition, Cisplatin, Fractionated radiation, Local tumor control, Tumor microenvironment, Human tumor xenograft

## Abstract

**Background:**

The transcription factor hypoxia-inducible factor-1 (HIF-1) pathway plays an important role in tumor response to cytotoxic treatments. We investigated the effects of a novel small molecule inhibitor of mitochondrial complex I and hypoxia-induced HIF-1 activity BAY-87-2243, on tumor microenvironment and response of human squamous cell carcinoma (hSCC) to clinically relevant fractionated radiotherapy (RT) with and without concomitant chemotherapy.

**Methods:**

When UT-SCC-5 hSCC xenografts in nude mice reached 6 mm in diameter BAY-87-2243 or carrier was administered before and/or during RT or radiochemotherapy with concomitant cisplatin (RCT). Local tumor control was evaluated 150 days after irradiation and the doses to control 50% of tumors (TCD_50_) were compared between treatment arms. Tumors were excised at different time points during BAY-87-2243 or carrier treatment for western blot and immunohistological investigations.

**Results:**

BAY-87-2243 markedly decreased nuclear HIF-1α expression and pimonidazole hypoxic fraction already after 3 days of drug treatment. BAY-87-2243 prior to RT significantly reduced TCD_50_ from 123 to 100 Gy (p=0.037). Additional BAY-87-2243 application during RT did not decrease TCD_50_. BAY-87-2243 before and during radiochemotherapy did not improve local tumor control.

**Conclusions:**

Pronounced reduction of tumor hypoxia by application of BAY-87-2243 prior to RT improved local tumor control. The results demonstrate that radiosensitizing effect importantly depends on treatment schedule. The data support further investigations of HIF-1 pathway inhibitors for radiotherapy and of predictive tests to select patients who will benefit from this combined treatment.

## Introduction

Many solid tumors express hypoxia-inducible factor-1α (HIF-1α), which is associated with poor prognosis after surgery, radiotherapy, and chemotherapy in several cancer types [1–5]. Tumor hypoxia, among other stress conditions [6–8], is recognized as a major regulator of multiple HIF-1-mediated pathways which promote cell survival [9]. Hypoxia leads to the stabilization and accumulation of HIF-1α protein, which translocates to the nucleus and forms a heterodimer with its partner HIF-1β. This transcriptional complex induces the transcription of numerous genes with adaptive functions, e.g. vascular endothelial growth factor and glucose transporter 1 to increase oxygen availability and to allow metabolic adaptation to oxygen deprivation.

Pharmacological or genetic targeting of HIF-1 sensitized tumor cells to radiation and chemotherapeutic DNA damaging agents and decreased tumor growth [10–15]. Beside direct radiosensitization of tumor cells caused by HIF-1 inhibition other mechanisms such as radiosensitization of tumor vasculature or reduction of tumor hypoxia have been shown to contribute to the enhanced effect of radiation therapy [16–19]. Resistance of hypoxic tumor cells to chemotherapy was attributed to several factors including poor drug distribution, reduced drug uptake, activation of genes leading to a drug-resistant phenotype [20]. Recent studies have demonstrated an important role of HIF-1 in resistance to chemotherapeutic agents such as platinum-containing anti-cancer drugs, e.g. through regulation of XPA (xeroderma pigmentosum group A) protein that senses DNA damage and recruits other DNA repair proteins to the damaged template in the nucleotide excision repair pathway [21, 22].

BAY-87-2243 inhibits mitochondrial production of reactive oxygen species (ROS) by blocking mitochondrial complex I, which subsequently reduces hypoxia-induced HIF-1 activity [23]. Being encouraged by our recent findings using the compound BAY-84-7296 with the same mode of action but lower on-target efficiency as its derivative BAY-87-2243, which completely resolved tumor hypoxia and pronouncedly increased local tumor control after irradiation with large single doses in two different hSCCs of head and neck, UT-SCC-14 and UT-SCC-5, *in vivo* [24], we tested in the present study whether BAY-87-2243 leads to the reduction of tumor hypoxia and improves the outcome of clinically relevant fractionated irradiation with and without concomitant cisplatin treatment. The fractionation protocol with 30 fractions over 6 weeks was chosen to account for potential interactions between the compound and radiobiological mechanisms of fractionated irradiation such as repopulation, reoxygenation, recovery and redistribution, which by design did not contribute to local tumor control after single dose irradiation. UT-SCC-5 hSCC was chosen for the experiments because this tumor model is more radioresistant and exhibits higher expression of HIF-1α and hypoxic fraction as compared with UT-SCC-14 [24, 25]. The efficacy of various combination regimens have been tested using a series of TCD_50_ (dose to cure 50% of tumors) assays in nude mice. We show that radiosensitizing effect of BAY-87-2243 with fractionated irradiation depends on treatment schedule, which may provide important information for the design of clinical trials.

## Methods

### BAY-87-2243, cisplatin

BAY-87-2243 was developed by Bayer Pharma AG. For the experiments BAY-87-2243 was dissolved in carrier solution (10% ethanol, 40% Solutol® HS15, 50% sterile distilled water) and administered orally by gavage (9 mg/kg/body weight [b.w.]). Control animals were treated with the carrier solution. Cisplatin (Calbiochem, Germany, 3 mg/kg/b.w.) dissolved in saline (NaCl 0.9%) was administered intraperitoneally (i.p.).

### Animals and tumor model

The experiments were performed using 7 to 14 week-old male and female NMRI (nu/nu) nude mice obtained from the pathogen-free animal breeding facility (Experimental Centre, Medical Faculty, TU Dresden, Germany). Two to five days prior to tumor transplantation the nude mice received a total body irradiation (4 Gy, 200 kV X-rays, 0.5 mm Cu-filter, ~1 Gy/min) for further immunosuppression. The experiments and the animal facilities were approved according to the institutional guidelines and the German animal welfare regulations.

The experiments were performed using the established hSCC of the tongue UT-SCC-5 with hypoxia-induced activation of HIF-1α [26]. Small pieces of a source tumor were transplanted subcutaneously into the right hind-leg of anesthetized mice (120 mg/kg b.w. ketamine i.p. and 16 mg/kg xylazine i.p.). The volume doubling time, histological examinations and DNA-microsatellite profile confirmed the identity of the transplanted UT-SCC-5 xenografts.

### Tumor sampling

BAY-87-2243 or carrier solution were applied once tumors reached 4 mm (~33 mm^3^) in diameter until the tumors reached 7 mm (~180 mm^3^) or once tumors reached 6 mm for 3, 5, or 7 days. At these time points the tumors were excised 24 h after the last carrier or BAY-87-2243 administration (3–9 mice per treatment group) for immunohistochemistry or western blotting. Prior to tumor harvesting the hypoxia marker pimonidazole (Natural Pharmacia International, Inc., Research Triangle Park, NC, USA; 0.1 mg/g b.w., dissolved at 10 mg/ml in 0.9% NaCl, i.p.) was injected one hour before excision as well as the perfusion marker Hoechst 33342 (Sigma Aldrich, Deisenhofen, Germany; 0.75 mg in PBS, intravenously [i.v.]) one minute before excision. Half of the tumor was immediately snap frozen in liquid nitrogen and stored at -80°C. The second half was fixed overnight in 4% formalin and embedded in paraffin.

### Tumor growth delay and TCD_50_ assay

In tumor growth delay assay randomized mice were treated daily with carrier (n=39) or BAY-87-2243 (n=41) when tumors reached a size of 4 mm in diameter until the tumors grew to 7 mm in diameter. Time to reach 7 mm was compared between treatment groups.

In TCD_50_ assay six experimental treatments were tested combining fractionated radiotherapy (RT) or cisplatin based radiochemotherapy (RCT) with BAY-87-2243:

1. RT: 30 fractions delivered over 6 weeks. Carrier was applied before and during radiotherapy.
2. BAY-87-2243 + RT: pre-treatment with BAY-87-2243 followed by RT with carrier.
3. BAY-87-2243 + RT/BAY-87-2243: treatment with BAY-87-2243 before and during RT.
4. RCT: 30 fractions over 6 weeks combined with cisplatin once per week after irradiation. Carrier was applied before and during RCT.
5. RCT/BAY-87-2243: pre-treatment with carrier followed by RCT with BAY-87-2243.
6. BAY-87-2243 + RCT/BAY-87-2243: treatment with BAY-87-2243 before and during RCT.

Pre-treatment with carrier or BAY-87-2243 was always performed on three consecutive days followed by RT 24 h after the last application. Carrier or BAY-87-2243 was administered after each radiation fraction during radiotherapy.

Treatments started when tumor size reached 6 mm in diameter. Animals were randomly allocated in six groups with total doses between 60 Gy and 160 Gy (5–12 mice per dose group) delivered in 30 equal fractions (dose per fraction between 2 and 5.3 Gy). All irradiations were performed under normal blood flow conditions without anaesthesia. During irradiation animals were immobilized using plastic tubes fixed on a lucite plate and the tumor-bearing leg was positioned in the irradiation field by a foot holder distal to the tumor. The tumor diameters were measured using a caliper twice per week and once per week 90 days after irradiation. Tumor volume was calculated by *π*/6 ⋅ *a* ⋅ *b*^2^, where a is the longest and b is the perpendicular shorter tumor diameter. When tumor volume increased for three consecutive measurements after shrinkage or when tumors continued to grow without shrinkage they were scored as recurrence. The animals were sacrificed when the recurrent tumor reached the diameter of 15 mm or when the animal appeared to suffer. A total of 343 mice were observed after treatment for a maximum time of 150 days, which is sufficient to detect all local failures in UT-SCC-5 [26]. Ninety five percent of 200 local failure events occurred before day 69 and the latest recurrence was scored at day 150. A total of 71 mice censored between 21 and 149 days were included in the analysis [25]. Fitting of the local control data, calculation of the TCD_50_ values, of 95% confidence intervals for TCD_50_ and of p-values for comparisons of treatment groups were performed as described previously [25]. Enhancement ratio (ER) was calculated as the ratio between the TCD_50_ value of the control group and the TCD_50_ of the experimental group.

### Western blot analysis of HIF-1α

Western blotting was performed one time according to the established protocol as described previously [24]. Protein samples were prepared using the NE-PER Nuclear and Cytoplasmic KIT (Thermo Scientific, Germany) according to the manufacturer’s instructions. Antibodies used were mouse monoclonal anti-human HIF-1α (1:250, BD Biosciences, USA) and rabbit polyclonal anti-histone-H2B (1:500, Imgenex, USA) and anti-Calpain 1 (1:1000, Cell Signaling, USA) served as the loading controls for nuclear or cytoplasmic cell compartments, respectively. Nuclear HIF-1α band intensities were normalized to histone-H2B levels.

### Histological studies

Two 10 μm frozen cross-sections from tumor centre were stained for pimonidazole (rabbit anti-pimonidazole antisera, Burlington, USA) and CD31 (rat anti-mouse CD31, clone MEC 13.3, PharMingen/BD Biosciences, Heidelberg, Germany), scanned and blindly analysed as described in detail previously [26]. After scanning, the same tumor sections were stained with haematoxylin and eosin for identification of viable and necrotic tumour subareas by morphological criteria. The pimonidazole hypoxic fraction (pHF) and the relative vascular area (RVA) were calculated as the percentage of the viable tumor area stained for pimonidazole or CD31, respectively. The fraction of perfused vessels (PF) was calculated as the percentage of the vascular area overlapped with Hoechst 33342 perfusion marker. Necrotic fraction (NF) was determined as the necrotic tumor area divided by the total tumor area. The immunohistochemistry staining protocol for HIF-1α (mouse monoclonal anti-human HIF-1α, BD Biosciences, USA) has previously been described in detail [24, 27].

### Statistical analysis

Normality of distributions was tested using the skewness and kurtosis test. Mean values were compared using the independent sample t-test. Non-parametric Mann–Whitney test was used to compare non-normal distributions. P-values were adjusted for multiple comparisons using the Scheffe correction when relevant. Statistical analysis was performed using a commercial software package STATA/SE 8.0 (STATA Corporation, College Station, TX, USA). P-values less or equal to 0.05 were considered as statistically significant.

## Results

**Figure 1:**
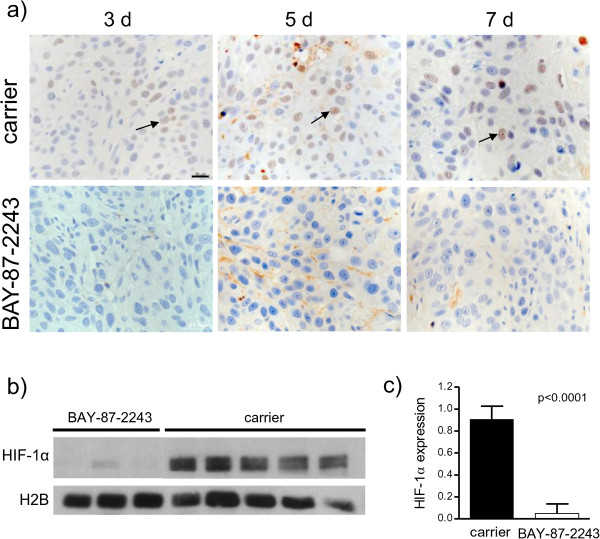
**BAY-87-2243 inhibits nuclear HIF-1α protein expression.** UT-SCC-5 tumors were treated either with BAY-87-2243 or carrier for 3, 5, or 7 days and were excised 24 h after end of treatment: **a)** immunohistochemistry, black arrows indicate the positive nuclear HIF-1α staining, scale bar: 20 μm; **b)** western blots of nuclear HIF-1α from different animals 3 days after BAY-87-2243 application; **c)** quantification of western blots by densitometry relative to histone-H2B, means ± SD.

**Figure 2:**
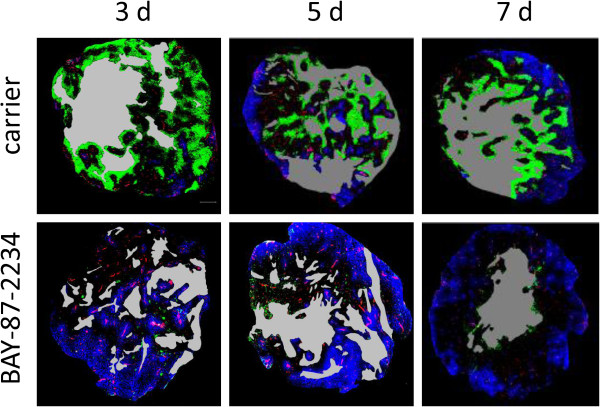
**Pseudo-coloured images of cross-sections from tumors excised 24 h after end of treatment with BAY-87-2243 or carrier for 3, 5 or 7 days.** Green: hypoxia, pimonidazole; blue: perfusion, Hoechst 33342; red: vascular endothelium, CD31; grey: necrotic areas. Scale bar: 500 μm.

**Figure 3:**
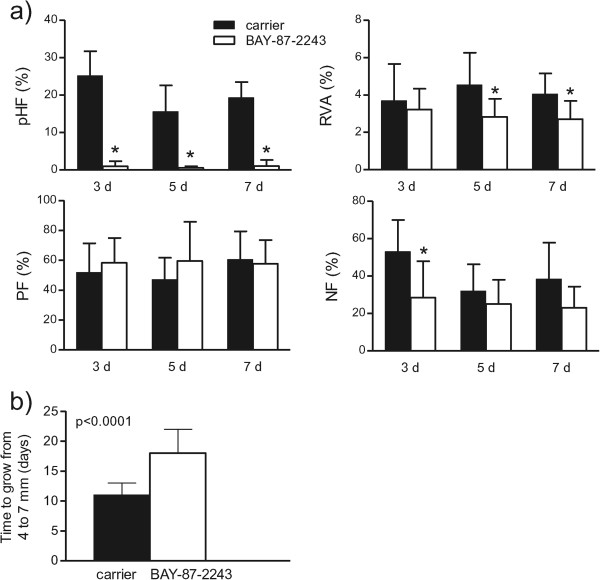
**Effects of BAY-87-2243 on tumor microenvironment and tumor growth. a)** Pimonidazole hypoxic fraction (pHF), relative vascular area (RVA), fraction of perfused vessels (PF), and necrotic fraction (NF) in UT-SCC-5 tumors 24 h after end of treatment with BAY-87-2243 or carrier for 3, 5, or 7 days (n=7-9 tumors per time point and treatment group). **b)** Time to grow from 4 to 7 mm tumor diameter. UT-SCC-5 tumors were treated daily with BAY-87-2243 or carrier. Bars represent means ± SD of histological parameters or medians ± MAD (median absolute deviation) of growth times. Asterisks indicate significant differences from carrier control.

To test the inhibitory effect of BAY-87-2243 on HIF-1α protein expression, tumors were treated with the drug for 3, 5 or 7 consecutive days. Nuclear HIF-1α protein levels were strongly suppressed after 3 days of drug treatment (Figure 1). Cytoplasmic cell extracts showed none or very weak HIF-1α protein expression (data not shown). To study the kinetics of changes in tumor microenvironment induced by BAY-87-2243, hypoxia, vasculature and perfused vessels were examined in tumors after 3, 5 and 7 days of drug treatment. Three daily applications of BAY-87-2243 markedly reduced pHF to 1% as compared with 25% in carrier-treated tumors (p < 0.0001, Figures 2 and 3a). This decrease in pHF remained after BAY-87-2243 treatment for 5 and 7 days. A statistically significant reduction in RVA was found 5 and 7 days after BAY-87-2243 treatment as compared with carrier-treated tumors (p=0.033 and p=0.026, respectively, Figure 3a). BAY-87-2243 did not affect PF at any time point. Necrotic fraction was statistically significantly lower only after 3 days of drug treatment (p=0.018).

Anti-tumor effects of BAY-87-2243 were tested using daily application from 4 mm tumor diameter to 7 mm. BAY-87-2243 alone significantly inhibited the growth of UT-SCC-5 tumors. The median time to reach 7 mm tumor diameter was 18 days for BAY-87-2243 as compared with 11 days for carrier treated tumors (p < 0.0001, Figure 3b). Administration of BAY-87-2243 for about 18 days significantly reduced HIF-1α protein expression (data not shown) as well as pHF (mean 2.4% (BAY-87-2243) vs. 17.6% (carrier), p < 0.0001), and NF (mean 9% vs. 35.6%, p=0.0002), whereas RVA and PF remained unchanged.

**Figure 4:**
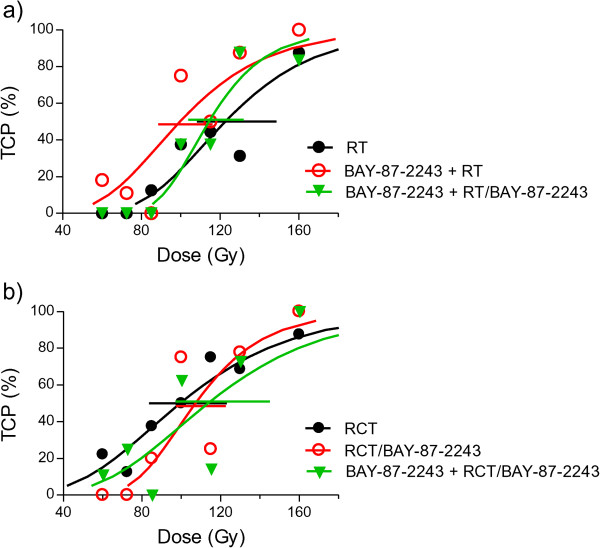
**Effect of BAY-87-2243 on local tumor control.** Dose-response curves after **a)** BAY-87-2243 combined with radiotherapy with 30 fractions within 6 weeks (RT) and **b)** BAY-87-2243 combined with fractionated radiochemotherapy with cisplatin (RCT). Symbols: observed local control rates; curves: calculated local tumor control probabilities (TCP); error bars: 95% CI of TCD50.

To test whether BAY-87-2243 improves local tumor control after clinically relevant fractionated irradiation (RT), the administration of BAY-87-2243 for 3 days was either halted before irradiation with 30 fractions within 6 weeks or was continued during irradiation. Whereas pre-treatment with BAY-87-2243 for 3 days prior to irradiation (BAY-87-2243 + RT) significantly reduced TCD_50_, the decrease in TCD_50_ after concomitant BAY-87-2243 treatment during irradiation (BAY-87-2243 + RT/BAY-87-2243) was less pronounced and statistically not significant (Table 1, Figure 4a). The enhancement ratio greater than 1 indicates that the dose to achieve the same local control rate (i.e. TCD_50_) is reduced by BAY-87-2243 in comparison with radiation alone. To test whether BAY-87-2243 decreases TCD_50_ after fractionated radiochemotherapy (RCT) BAY-87-2243 was combined with cisplatin and irradiation with 30 fractions within 6 weeks. The TCD_50_ after RCT was lower by a factor of 1.23 in comparison with RT alone. This effect was only marginally significant (p=0.09) likely due to considerable intertumoral heterogeneity in response to RCT as suggested by shallow dose–response curve (Figure 4b). Local tumor control was not significantly improved if BAY-87-2243 was administered during fractionated irradiation combined with weekly administration of cisplatin (RCT/BAY-87-2243). Pre-treatment with BAY-87-2243 for 3 days in addition to continuous BAY-87-2243 application during RCT (BAY-87-2243 + RCT/BAY-87-2243) also did not result in reduction of TCD_50_ (Table 1, Figure 4b).

**Table 1:** **Summary of TCD**_**50**_ **values and enhancement ratios (ER) for different treatments**

Experimental arm	TCD _50_[95% CI] (Gy) (p-value vs. control)	ER
1) RT	122.7 [108;149] -	-
2) BAY-87-2243 + RT	99.7 [88;117] (0.037)	1.23
3) BAY-87-2243 + RT/BAY-87-2243	114.3 [103;132] (0.4)	1.07
4) RCT	99.5 [83;124] -	-
5) RCT/BAY-87-2243	107.2 [97;123] (0.5)	0.93
6) BAY-87-2243 + RCT/BAY-87-2243	113.6 [97; 145] (0.3)	0.88

P-value <0.05 indicates significant differences between experimental treatment and respective control (RT or RCT).

## Discussion

To our knowledge this experimental study demonstrates for the first time that BAY-87-2243, an inhibitor of hypoxia-induced gene activation, improves local tumor control after clinically relevant fractionated irradiation. The novelty of our findings is given by the fact that here we used fractionated irradiation which, in contrast to single dose irradiation used in our previous experiment [24], allows for specific scheduling evaluation and potential interaction with radiobiological mechanisms relevant to local tumor control after fractionated irradiation such as reoxygenation, repopulation, recovery and redistribution. The radiosensitizing effect was schedule-dependent and only significant if BAY-87-2243 was administered prior to fractionated irradiation. In contrast, concomitant administration of BAY-87-2243 during fractionated irradiation with or without cisplatin did not improve local tumor control. Whether pre-treatment with BAY-87-2243 before start of radiochemotherapy affects local tumor control was not tested in the present experiments and remains to be elucidated. Thus, our data do not allow to assess whether the efficacy of BAY-87-2243 before initiation of fractionated radiotherapy depends on whether this is given alone or in combination with chemotherapy. When the experiments were designed we hypothesized based on our previous data [24] and findings by others that experimental arms 3 (BAY-87-2243 + RT/BAY-87-2243) and 6 (BAY-87-2243 + RCT/BAY-87-2243) are the most effective ones. However, unexpectedly concomitant BAY-87-2243 was not effective or even reversed the effect of pre-RT drug administration. Taken together, our data indicate a schedule dependence of the radiosensitizing effect of BAY-87-2243 but further experiments are necessary to confirm this conclusion.

The present data and our previous findings [24] suggest that the reduction in tumor hypoxia represents the underlying radiosensitizing mechanism of BAY-87-2243. Although we did not specifically investigate the mechanisms of reoxygenation induced by BAY-87-2243, the observed rapid drop in hypoxia already after 3 days of treatment with unchanged tumor vascular area and perfused functional vessels may suggest a cytotoxic effect of BAY-87-2243 on hypoxic tumor cells, e.g. blockage of HIF-1-mediated hypoxia tolerance [28]. However, necrotic fraction initially decreased and later remained unchanged in BAY-87-2243 treated tumors, it remains unclear to what extent rapid resorption of necrotic cells and/or reduction of proliferation and thereby oxygen consumption may have contributed to this observation. Normalisation of the microvasculature appears less likely to explain the reduction in hypoxia as longer treatments with BAY-87-2243 for 5 and 7 days resulted in a decrease of vascular area and unchanged perfused vessels. Reduction in tumor vascularization upon HIF-1 inhibition has been also demonstrated by others, which was associated with the decreased gene expression of angiogenic growth factors and decreased mobilization of circulating angiogenic cells [29]. In the latter study these effects on tumor vasculature contributed to the reduced growth of tumor xenografts similarly to our findings. Another potential mechanism by which BAY-87-2243 reduces hypoxic fraction such as inhibition of mitochondrial oxygen consumption [23, 30] appears rather less likely because UT-SCC-5 tumors exhibit very high glycolytic activity *in vivo* [31–33].

In our previous study pre-treatment with BAY-84-7296 for two weeks radiosensitized tumors to irradiation with large single doses resulting in an enhancement ratio of 1.37 [24], which indicates that the dose to achieve the same level of local tumor control could be reduced by a factor of 1.37 if tumors are pre-treated with the inhibitor. This significant radiosensitizing effect could be explained at least to some extent by the pronounced reduction of the fraction of radioresistant hypoxic tumor cells at time of irradiation. These previous findings were obtained using BAY-84-7296 and prompted us to evaluate here the effect of its derivative BAY-87-2243 on local tumor control after clinically relevant fractionated irradiation. BAY-87-2243 was chosen due to its higher on-target activity for further experimental and clinical evaluation. The lead compound BAY-84-7296 was discovered by a small-molecule library screen for its activity to suppress HIF-1α protein accumulation under hypoxic conditions. Subsequent studies on BAY-87-2243 revealed the mode of action, i.e. inhibition of mitochondrial complex I and reduced hypoxia-induced HIF-1 activity [23]. In the present study pre-treatment time with BAY-87-2243 was reduced to 3 days because we showed that shorter treatment is sufficient to resolve tumor hypoxia as detected by pimonidazole hypoxia marker. Pre-treatment with BAY-87-2243 resulted in an enhancement ratio of 1.23. This effect was less pronounced in comparison with ER of 1.37 obtained after large single radiation doses and BAY-84-7296. In addition to longer pre-treatment time with the compound in single dose experiment, several biological factors may contribute to this difference. First, radiation response after single doses may depend on the hypoxic fraction to a greater extent than the outcome of fractionated irradiation leading to a more pronounced radiosensitizing effect in a single dose setting. Extensive reoxygenation between radiation fractions in UT-SCC-5 [25] may at least in part explain lower ER. Second, it is well established that in addition to hypoxia other radiobiological mechanisms such as repopulation, repair, etc. determine tumor response to fractionated irradiation, which can attenuate the effect of the compound [34].

It has been shown that several other mechanisms can contribute to the enhanced tumour radiation response in combination with HIF-1 inhibition. This includes prevention of reconstitution of stromal function after radiation and further sensitization of tumour vessels to radiation damage upon HIF-1 inhibition [10, 17, 18]. These findings together with the data showing a radioprotective function of HIF-1α in accumulation of DNA damage response proteins [35] led us to hypothesize that application of BAY-87-2243 during fractionated irradiation in addition to pre-treatment prior to radiotherapy further improves local tumor control. Despite of these potential mechanistic advantages, the latter treatment scheme however, was less efficacious than pre-treatment with BAY-87-2243 and resulted in an ER of only 1.07. The underlying reasons remain unclear. HIF-1α expression in the present study markedly decreased during BAY-87-2243 treatment prior to irradiation and it was not tested here whether HIF-1α recovered thereafter during irradiation to potentiate the effect of BAY-87-2243. However, we have previously shown that in UT-SCC-5 tumors despite of extensive reoxygenation during fractionated irradiation the levels of HIF-1α remained unchanged [27] as opposed to HIF-1 up-regulation detected by others in other tumor cell lines [6, 8, 16]. Based on these findings, it may be hypothesised that limited expression of the molecular target during the course of radiotherapy underlies the lack of further improvement of local control. In addition it cannot be excluded that BAY-87-2243 during irradiation might induce adverse vascular effects with potentially negative consequences for perfusion and reoxygenation which may have attenuated the advantageous effect of pre-treatment with BAY-87-2243. Importantly, our finding that BAY-87-2243 is efficacious when given before but not during or after RT is in contrast to several other reports [16, 17, 36]. In the latter studies which used tumor growth delay as experimental endpoint, HIF-1 inhibition during and after RT but not prior to RT was efficacious. Whereas HIF-1 inhibition during radiotherapy may decrease the growth rate of surviving tumor cells influencing growth delay, it may not induce killing of tumor stem cells to an extent detectable as improved local tumor control. In addition one may speculate that the pharmacological effect of BAY-87-2243 on mitochondrial production of ROS and subsequent lower HIF-1 levels may have different radiobiological consequences than other HIF-1 targeting drugs or genetic knock-down.

Experimental evidence is accumulating that supports an important role of HIF-1 in chemoresistance by multiple mechanisms including increased drug efflux and/or decreased drug uptake, alteration of drug targets, cell cycle arrest in G1 affecting the cytotoxicity of cell phase-specific agents, etc. [37]. Deficiencies in nucleotide excision repair and in mismatch repair negatively affect the cytotoxicity of DNA interacting agents such as cisplatin [38]. HIF-1 has been shown in pre-clinical studies to interfere with these repair systems and it therefore has been suggested that HIF-1 inhibition can improve the outcome of radiochemotherapy [14, 21, 22, 39]. In support of this hypothesis a recent clinical study has revealed a negative correlation between pre-treatment HIF-1α expression in head and neck tumors and overall survival after concomitant cisplatin-based radiochemotherapy [40]. In line with other pre-clinical and clinical studies, our results show marginally significant enhancement of the radiation response by cisplatin [41–44]. BAY-87-2243 administered to tumor-bearing animals before and during fractionated cisplatin-based radiochemotherapy did not improve local tumor control in a single tumor hSCC. The mechanisms underlying the potential interactions between two different compounds and radiation and their net effect on local control have not been addressed in this study and need further investigations. Classical experiments have been reported that cisplatin showed greater enhancement in fully hypoxic (clamped) tumors than in tumors containing both hypoxic and well oxygenated cells (unclamped) [42]. It can be therefore speculated that in the present study pronounced reduction of hypoxic fraction by BAY-87-2243 might at least in part counteract the effect of cisplatin. It is however important to take into account considerable intertumoral heterogeneity in response to radiochemotherapy in the present study, which limits the interpretation of these data. Nevertheless, from a clinical point of view our results might have important implications for further development of this compound in the context of fractionated radiotherapy. While for radiochemotherapy it appears that caution and further experiments are needed, combination of compounds like BAY-87-2243 with radiation alone is of great clinical interest because many patients are medically unfit for concurrent radiochemotherapy. In addition, classical chemotherapy has substantial side effects and might exhibit only a limited benefit over radiotherapy alone with regard to local tumor control.

## Conclusion

Pronounced reduction of tumor hypoxia by application of the novel inhibitor of hypoxia-induced gene activation BAY-87-2243 before start of fractionated radiotherapy improved local tumor control. The therapeutic effect of BAY-87-2243 importantly depended on treatment schedule and was only significant if BAY-87-2243 was administered prior to fractionated radiotherapy. The data support the further investigation of such inhibitors for combination with radiotherapy. In particular further radiobiological studies are needed to better understand the interaction of BAY-87-2243 with radiotherapy and radiochemotherapy which is a prerequisite for the development of predictive tests and tools for monitoring.

## Authors’ information

AY and DZ - share senior authorship.
